# Correction: LRP1 integrates murine macrophage cholesterol homeostasis and inflammatory responses in atherosclerosis

**DOI:** 10.7554/eLife.104437

**Published:** 2024-10-15

**Authors:** Xunde Xian, Yinyuan Ding, Marco Dieckmann, Li Zhou, Florian Plattner, Mingxia Liu, John S Parks, Robert E Hammer, Philippe Boucher, Shirling Tsai, Joachim Herz

**Keywords:** Mouse

Xian X, Ding Y, Dieckmann M, Zhou L, Plattner F, Liu M, Parks JS, Hammer RE, Boucher P, Tsai S, Herz J. 2017. LRP1 integrates murine macrophage cholesterol homeostasis and inflammatory responses in atherosclerosis. *eLife* **6**:e29292. doi: 10.7554/eLife.29292.

Published 16 November 2017

It was brought to our attention via PubPeer that the protein bands of Akt and β -actin in Figure 4C are identical.

This unfortunate duplication of β -actin occurred during panel assembly due to the similar signal pattern of the relevant proteins, i.e. no change in expression of β-actin or Akt across treatments or genotypes. We have replaced the inadvertently duplicated β-actin blot with the correct immunoblot depicting Akt expression obtained in the same experiment.

As shown in the corrected Figure 4C, there are no differences in total Akt levels between genotypes before and after PDGF-BB treatment. Thus, the conclusions of this experiment, as well as the legend of Figure 4C remain unchanged.

We sincerely apologize for this inadvertent mistake during figure assembly.

The corrected Figure 4C is shown here:

**Figure 1:**
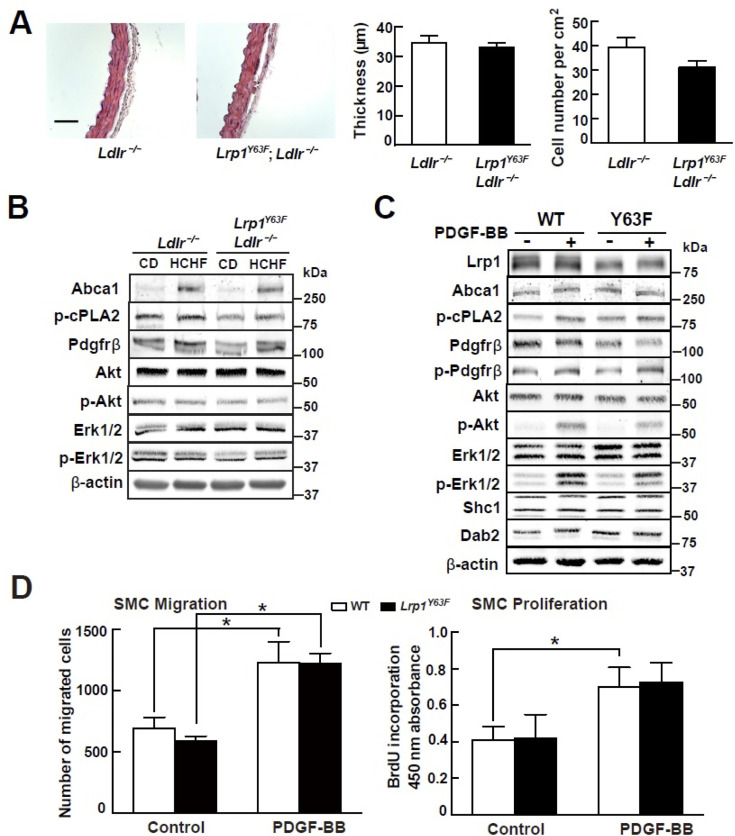


The originally published Figure 4 is shown here as reference:

**Figure 2:**
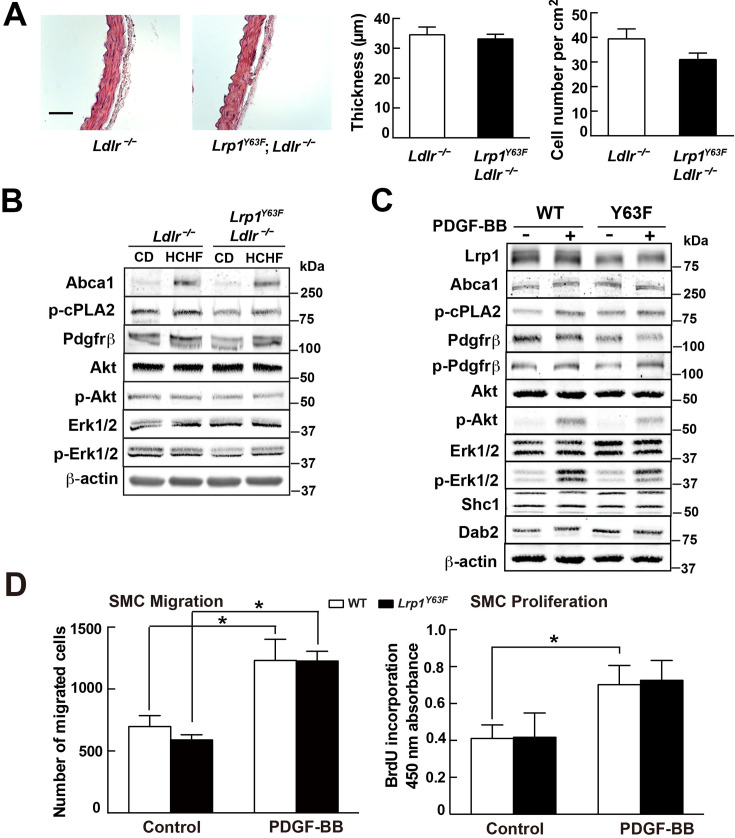


We thank the users of PubPeer for alerting us to this issue.

The article has been corrected accordingly.

